# Arbitrarily Large Area Graphene Suspension with Ultralow Standoff for Varying Capacitance Applications

**DOI:** 10.3390/nano16090565

**Published:** 2026-05-03

**Authors:** Tamzeed B. Amin, Md R. Kabir, Syed M. Rahman, Ashaduzzaman, James M. Mangum, Paul M. Thibado

**Affiliations:** 1 Department of Physics, University of Arkansas, Fayetteville, AR 72701, USA; tbamin@uark.edu (T.B.A.); jmmangum@uark.edu (J.M.M.); 2 Materials Science and Engineering Program, University of Arkansas, Fayetteville, AR 72701, USA; kabir@uark.edu (M.R.K.); sr096@uark.edu (S.M.R.); a004@uark.edu (A.)

**Keywords:** graphene, wet etching, atomic force microscopy, graphene transfer, critical point drying, wire bonding, variable capacitor

## Abstract

Freestanding graphene exhibits exceptional mechanical flexibility and electrical conductivity, making it well suited for varying capacitance applications. For example, when suspended above a fixed electrode, graphene will move in response to an applied bias voltage, thereby forming a varactor or voltage-controlled capacitor. In this work, we present a very detailed and scalable fabrication process for building graphene-based variable capacitor device structures. Starting with commercially available 100 mm silicon wafers with a thick thermal oxide layer, we fabricate thousands of individually accessible freestanding graphene variable capacitors using standard semiconductor methods. The process begins with metal deposition to establish alignment crosshairs, then oxide etching to create trenches, a second metal deposition to form electrodes and bonding pads, followed by large-area graphene transfer, then patterning the graphene via oxygen plasma etching, critical point drying for suspension, and finally wire bonding our devices into a package. We use optical and atomic force microscopy characterization to confirm our design specifications were met. Electrical characterization confirms successful graphene suspension through voltage-dependent capacitance measurements. The procedure presented here successfully suspends both pure multilayer graphene as well as graphene with a thick layer of PMMA.

## 1. Introduction

Recent developments in ultralow power electronics have markedly reduced the power requirements of sensor systems to the microwatt range during active mode and to the nanowatt range in standby mode [1,2,3,4]. For applications with low duty cycles, the average power consumption is only marginally higher than standby mode [5]. These remarkable breakthroughs have enabled the possibility of powering sensors without batteries and instead harvesting its power needs from the local environment. Readily available power sources include temperature gradients, mechanical vibrations, and light [6,7,8,9,10,11].

Initial designs for mechanical energy harvesting used a varying capacitance system as an electrostatic generator [12]. Subsequent advancements demonstrated the viability of microelectromechanical systems (MEMSs) utilizing electrostatic and piezoelectric properties to harvest energy from environmental vibrations [8,13,14,15].

Due to its novel properties, graphene is a strong candidate for use in low-power energy harvesting systems. For example, graphene is an atomically thin, very flexible, and optically transparent [16,17,18,19,20]. It is also very durable with the highest tensile strength [21], and a very high Young’s modulus [22,23,24,25]. It is also metallic [26,27,28]. When doped, it is ferromagnetic at room temperature [29,30]. Additionally, the number of atomic layers in a graphene sheet can be tuned based on growth conditions, which affects all of these fundamental properties [31,32,33,34].

Graphene’s metallic yet transparent properties have already proven useful for solar energy harvesting. When an interface is created between graphene and silicon a Schottky barrier naturally forms [35]. Thus when light passes through graphene a photocurrent is generated. Numerous studies have demonstrated the efficacy of graphene for fabricating Schottky solar cells and characterized the role of graphene thickness, doping, and surface texture [36,37,38,39,40,41,42,43]. Our group has also developed a fabrication process for graphene solar cells, and it purposely mimics the strategy presented here [44]. This allows for the potential fabrication of a single graphene device that can harvest both solar and kinetic energy. Our group also demonstrated that graphene solar cells can power a sensor without using batteries [45].

The usefulness of graphene’s two-dimensional nature extends beyond its ability to be transferred onto a rigid silicon surface for solar cell fabrication. When made freestanding, graphene naturally self-compresses to form an array of ripples, which was famously thought to be impossible [46]. Ripples in graphene have been extensively studied with theoretical methods such as kinetic Ising models [47,48], Monte Carlo [49,50], density functional theory [51,52], and molecular dynamics [53,54]. Graphene ripples have also been heavily experimentally studied using scanning tunneling microscopy [55,56], atomic force microscopy (AFM) [57,58], and transmission electron microscopy [59].

From all the above studies and others, we know that graphene ripples are in constant motion [60,61,62]. Few-layer graphene has a thickness of about 1 nm and, hence, is extremely flexible [63,64,65,66]. For comparison, the thinnest cantilevers, which are designed to fluctuate in response to stimuli, have a beam thickness of about 10 nm [67]. As a result, the flexural rigidity of graphene is about three orders of magnitude lower than these cantilevers [68,69]. Flexural rigidity not only depends on thickness, but also on Young’s modulus and Poisson’s ratio. The responsiveness of freestanding graphene to its environment has allowed it to be used for thermal sensing [70,71], liquid pressure sensors [72,73], and chemical sensors [74,75]. Since freestanding graphene is in constant motion, when near a fixed electrode the pair acts as a variable capacitor being driven by local vibrations, which can be used for energy harvesting [76,77,78].

Recent advances in chemical vapor deposition graphene growth and transfer methods have enabled the fabrication of ultra-large-area suspended graphene membranes. PMMA-assisted transfers have been used to prepare high-quality monolayer and bilayer graphene membranes on through-hole substrates with diameters ranging from 10 to 750 μm [79,80]. Further improvements, including bubbling transfer, solvent replacement, and sublimation-assisted methods, have enabled suspended graphene structures with lateral dimensions extending to the millimeter scale and with high yield [81,82]. It is more difficult, but some groups have suspended large-area graphene over closed cavities [83]. For varying capacitance applications, what is needed is ultra-large area graphene suspension with an ultralow standoff.

**Figure 1:**
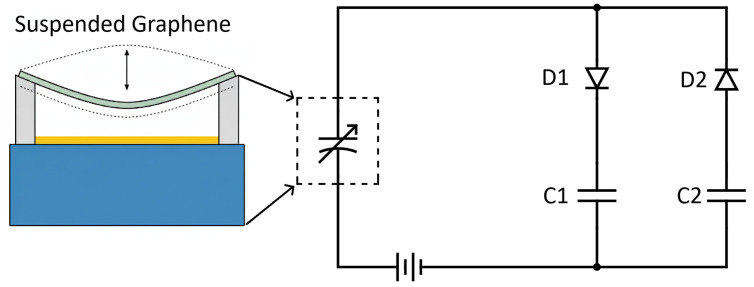
Illustration of a graphene variable capacitor and an electrical schematic of an energy harvesting circuit consisting of two diodes, two storage capacitors, a graphene variable capacitor, and a fixed DC bias voltage.

Besides the varactor, another fascinating varying capacitance application is energy harvesting as illustrated in Figure 1. The graphene variable capacitor is on the left branch. When the graphene membrane vibrates, the separation between the membrane and the fixed electrode changes over time, which causes the capacitance to vary. A fixed DC bias voltage *V* and a time-dependent capacitance C(t), will force electrical charges q(t) to flow on and off the graphene capacitor according to the relation q(t)=C(t)V. When charges flow clockwise, they pass through diode D1 and charge storage capacitor C1. When charges flow counterclockwise, they will recharge the DC bias, add charge to C2, and pass through diode D2. In this way, the mechanical motion of the graphene membrane is converted to stored electrical charge. Our group recently demonstrated that a mechanically rotated variable capacitor can power a sensor. The circuit performance was extensively characterized in terms of the capacitor rotation frequency, *f* the capacitance variation, ΔC and the magnitude of the DC bias voltage, *V* [84]. A simple formula was found for the average rectified current to be 2VΔCf, and, in addition, we demonstrated that only 30 nA is required to power the latest ultralow power consuming sensors mentioned earlier.

In this study, we fabricate graphene variable capacitors by creating trenches in the oxide-coated silicon wafer, positioning fixed electrodes at the bottom of each trench, and suspending graphene over the trenches. Our group successfully suspended graphene over a trench in an earlier study [77]. However, the goal here is to increase the total area of the suspended graphene to an arbitrarily large value. To achieve this the process had to be revisited, improved, and additional steps had to be added. We present the fabrication steps in this paper as follows. We begin by depositing gold alignment markers on the surface oxide to maintain the device orientation throughout the process. We then create an array of trenches by etching away part of the oxide coating. We deposit more gold to create bonding pads attached to long metal traces at the bottom of each trench. We also create four additional bonding pads and long traces outside the trenches to create our graphene electrical contact. We transfer a sheet of graphene so it is suspended above the gold traces in the trenches but also rests on the graphene electrical contact trace. The graphene sheet is then plasma etched to remove graphene from alternating regions over the trenches to aid suspension. The final device is wire bonded into a 28-pin package and tested. The capacitance is measured using plus and minus DC bias voltages to confirm the graphene deflects with increasing electrostatic force.

## 2. Materials and Methods

**Figure 2:**
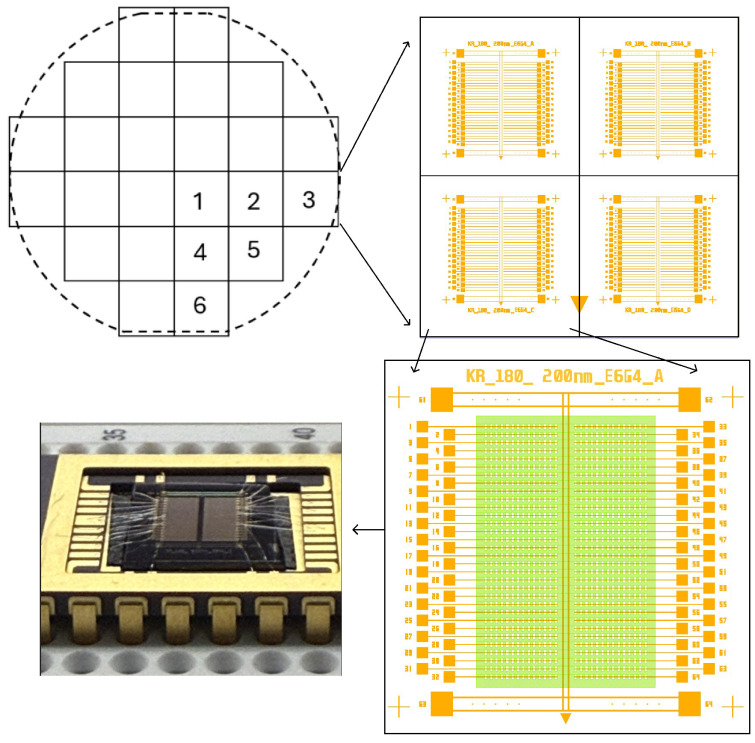
Wafer-to-package concept. The (**top-left**) schematic shows a 100 mm wafer can be diced into 24 sections or chips. The (**top-right**) schematic shows four devices prepared in parallel for each of the 24 chips. The (**bottom-right**) schematic shows a single device with the placement location for a rectangular sheet of graphene on top in green. The (**bottom-left**) photograph shows the completed device wire-bonded into a 28-pin package for electrical testing.

Commercially available 100 mm silicon wafers with 1–10 Ω·cm resistivity, 500 μm thickness, 〈100〉 orientation, and a 2 μm wet thermal oxide layer were used in this study. The wafer was first divided into four quadrants, and then each quadrant was further diced into six chips, as shown in the top left of Figure 2 and labeled 1 through 6. Each chip measures 16.6 mm × 16.6 mm and will eventually contain four identical devices prepared in parallel, as illustrated in the top right of Figure 2. After fabrication and dicing, multilayer graphene is placed on each device as shown in green in the bottom right of Figure 2. The completed device is wire-bonded into a standard 28-pin package as shown in the bottom left of Figure 2.

## 3. Results and Discussion

### 3.1. Alignment Markers

#### 3.1.1. Alignment Marker Design

**Figure 3:**
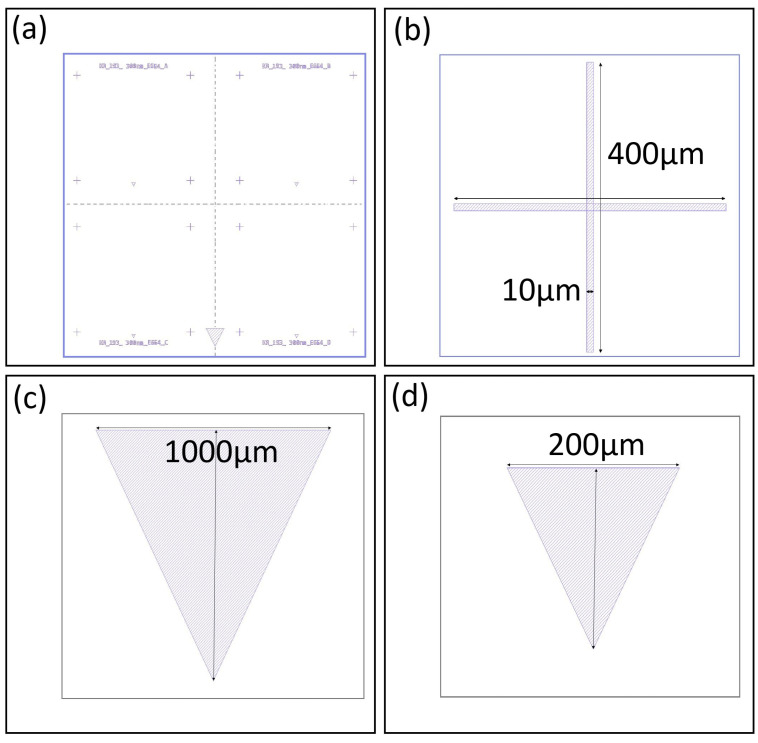
Alignment marker design. (**a**) Full-chip alignment pattern showing four identical devices and one large triangle at the bottom of the chip. Each device has a label, four crosshairs, and one small triangle. (**b**) Zoomed-in view of a crosshair with its dimensions labeled. (**c**) Zoomed-in view of our single large triangle with its dimensions labeled. (**d**) Zoomed-in view of our small triangle with its dimensions labeled.

The first step in our process is to create our alignment marker design, as shown in Figure 3a. This pattern shows four devices made in parallel as mentioned earlier. Each device contains a label, four crosshair markers, and one small triangle marker. In addition, the full-chip pattern includes one large triangle marker at the bottom. A zoomed in view of one crosshair is shown in Figure 3b. It has two perpendicular rectangles measuring 400 μm by 10 μm. A zoomed in view of the single large triangle is shown in Figure 3c. It has a base and height of 1000 μm. A zoomed in view of a smaller triangle marker is shown in Figure 3d. It has a base and height of 200 μm. In general, these markers allow us to precisely align the chips into the various instruments and to align one layer to another.

#### 3.1.2. Alignment Marker Process

**Figure 4:**
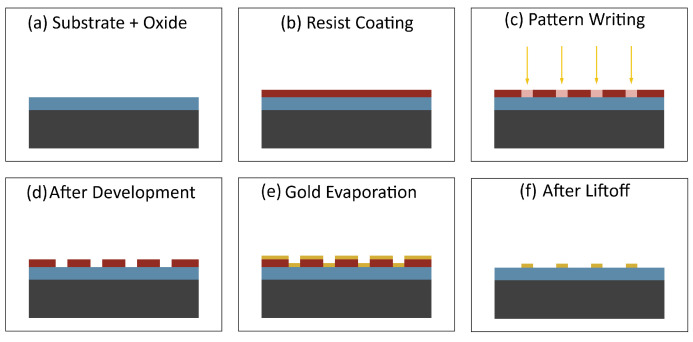
Alignment marker process. (**a**) Clean chip, (**b**) add photoresist, (**c**) write pattern, (**d**) develop, (**e**) add chromium then gold, and (**f**) lift-off unexposed photoresist.

An illustration of our alignment marker fabrication process is shown in Figure 4. The process begins with a clean and dry chip as illustrated in Figure 4a. Cleaning the chip involves acetone ultrasonic for 5 min, IPA ultrasonic for 5 min, DI water ultrasonic for 5 min, dry with compressed nitrogen, anneal at 180 °C for 15 min, and plasma etch for 1 min in $O_{2}$. A photoresist layer is then added as shown in Figure 4b. The chip is spin-coated at 2500 rpm for 1 min and baked at 110 °C for 3 min. Our alignment pattern is transferred onto the photoresist using maskless photolithography, as shown in Figure 4c. The chip is developed for 30 s, followed by rinsing in DI water for 1 min and the result is illustrated in Figure 4d. Next 5 nm of chromium followed by 45 nm of gold is deposited as illustrated in Figure 4e. Finally, lift-off is performed to remove the unexposed photoresist leaving only our metal pattern as illustrated in Figure 4f. Removing the unexposed photoresist involves stripper soak at 80 °C for 10 min, second stripper soak at 80 °C for 10 min, IPA ultrasonic for 5 min, DI water ultrasonic for 5 min, dry with compressed nitrogen, anneal at 180 °C for 15 min, and plasma etch for 1 min in $O_{2}$.

#### 3.1.3. Alignment Marker Characterization

**Figure 5:**
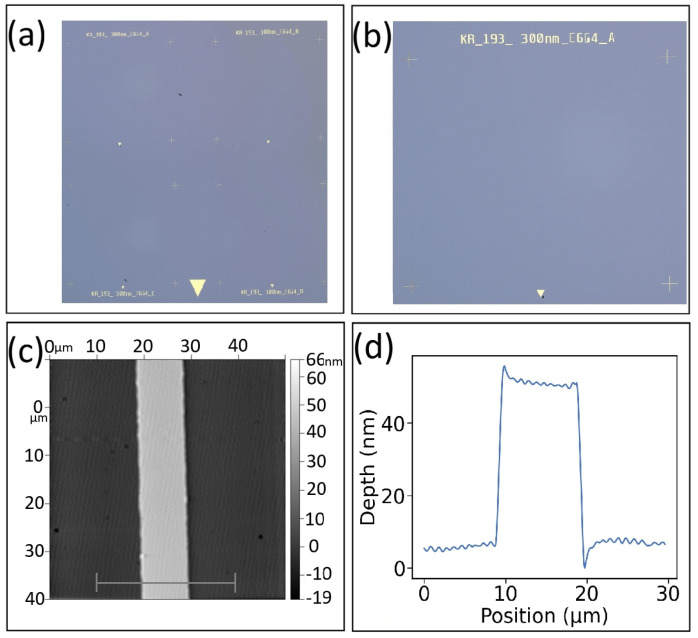
Alignment marker characterization. (**a**) Optical image of the full chip, (**b**) optical image of one device, (**c**) AFM image of crosshair trace, and (**d**) AFM line profile showing the crosshair trace width and thickness.

An optical image of our full chip after the metal alignment pattern is transferred is shown in Figure 5a. The four sample labels and the large triangle are relatively easy to see at this scale. A higher-magnification image of a single device on this same chip is shown in Figure 5b. The sample label, the four crosshairs, and the small triangle are visible. An AFM image of a section of a crosshair is shown in Figure 5c. AFM measurements are used to determine the achieved crosshair width and thickness. A line profile taken from the AFM image is shown in Figure 5d. The crosshair is about 10 μm wide and 50 nm thick. This metal thickness is enough for the maskless photolithography automatic alignment feature to detect. The large and small triangles are used by the person placing the chip into the maskless photolithography system.

### 3.2. Oxide Etch

#### 3.2.1. Oxide Etch Pattern Design

**Figure 6:**
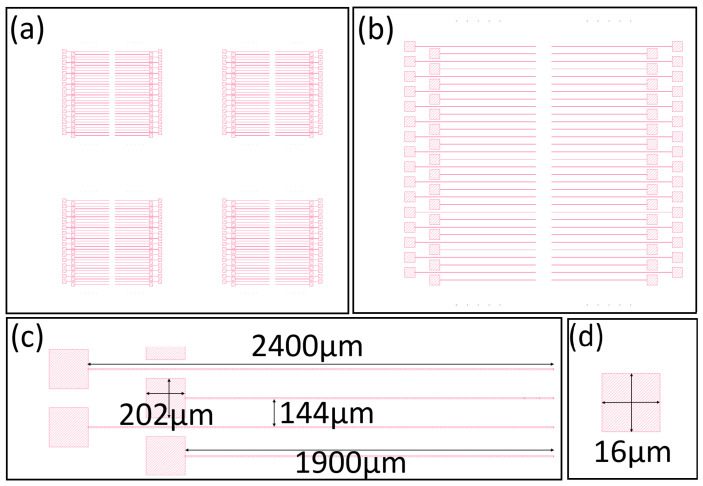
Oxide etch pattern design. (**a**) Full-chip etch pattern design showing four identical devices. (**b**) Zoomed-in view of one device showing 64 trenches. (**c**) Zoomed-in view of 4 trenches with their dimensions. (**d**) Zoomed-in view of one small square used for etch diagnostics with its dimensions.

The second step in our process is to create the oxide etch pattern design, as shown in Figure 6a. Again, the pattern creates four devices in parallel. A zoomed-in view of one device is shown in Figure 6b. Here it is easy to see that each device has 64 trenches with large square pads at one end. Above and below the array of trenches is an array of small squares. A zoomed in view of four trenches is shown in Figure 6c. The trenches are 6 μm wide and around 2000 μm in length. At the end of each trench is a large square measuring 200 μm on each side. This large square is where the bonding pad will be built. Notice we staggered the bonding pads to make more space for the wire bonder. A zoomed-in view of one of the smaller squares measuring 16 μm on each side is shown in Figure 6d. The small squares are for diagnostics and its function will be discussed later.

#### 3.2.2. Oxide Etch Process

**Figure 7:**
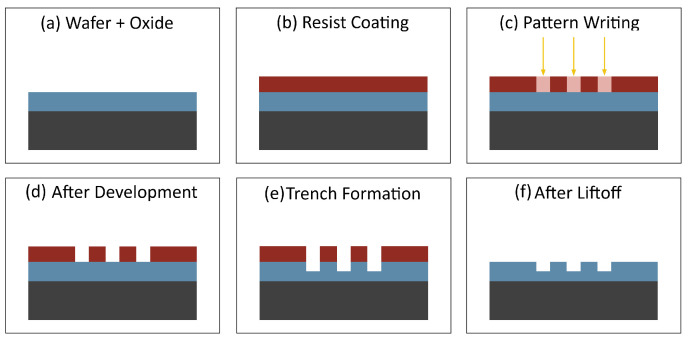
Oxide etch process. (**a**) Clean chip, (**b**) add photoresist, (**c**) write pattern, (**d**) develop, (**e**) oxide etch, and (**f**) lift-off unexposed photoresist.

An illustration of our chip oxide etch process is shown in Figure 7. The process begins with a clean and dry chip as shown in Figure 7a. This chip has the gold alignment markers on it. We follow the same chip cleaning procedure discussed earlier. A photoresist layer is then added as shown in Figure 7b. We follow the same procedure discussed earlier. Our etch pattern is transferred to the photoresist using a maskless photolithography system, as shown in Figure 7c. The chip is then developed as before to reveal the pattern as shown in Figure 7d. The chip is then etched as shown in Figure 7e. The etch process involves placing the chip in a 50:1 buffered oxide etch solution which removes the exposed $SiO_{2}$ until the desired depth is achieved. Finally, lift-off is performed using the same procedure as before leaving our etched pattern in the chip, as shown in Figure 7f.

#### 3.2.3. Oxide Etch Characterization

**Figure 8:**
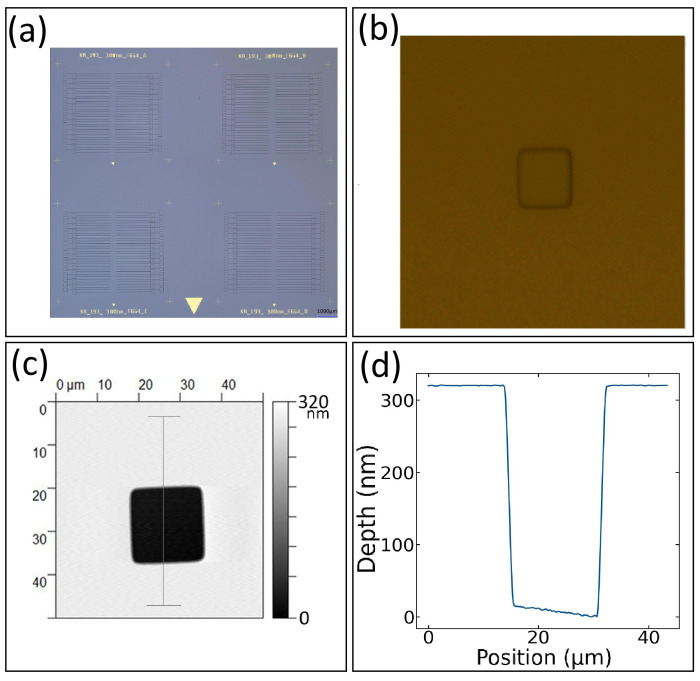
Oxide etch characterization. (**a**) Optical image of the full chip, (**b**) optical image of one small square etched into a deep well, (**c**) AFM image of one small square etched into a deep well, and (**d**) an AFM line profile showing the achieved width and depth of one small etched deep well.

An optical image of our chip after etching is shown in Figure 8a. The four identical etch patterns are faint. Nevertheless, one can make out that the pattern was successfully etched into the chip. A high magnification optical image of our small square is shown in Figure 8b. An AFM image of the same square is shown in Figure 8c. A line profile taken from the AFM image is shown in Figure 8d. The small square was etched into a deep well and it is about 16 μm across. More importantly, the depth of etched well was found to be about 300 nm using AFM. This is the same etch depth achieved throughout the chip. This depth plays a critical role in the stand-off between the top surface of the chip where the graphene will be placed and the bottom of the trench. Of course, we still need to deposit a metal trace at the bottom of the trench. We do have some control over the thickness of the metal layer we deposit. As a result, next we use the thickness of the metal layer to fine tune the stand-off height.

### 3.3. Metal Deposition

#### 3.3.1. Metal Deposition Pattern

**Figure 9:**
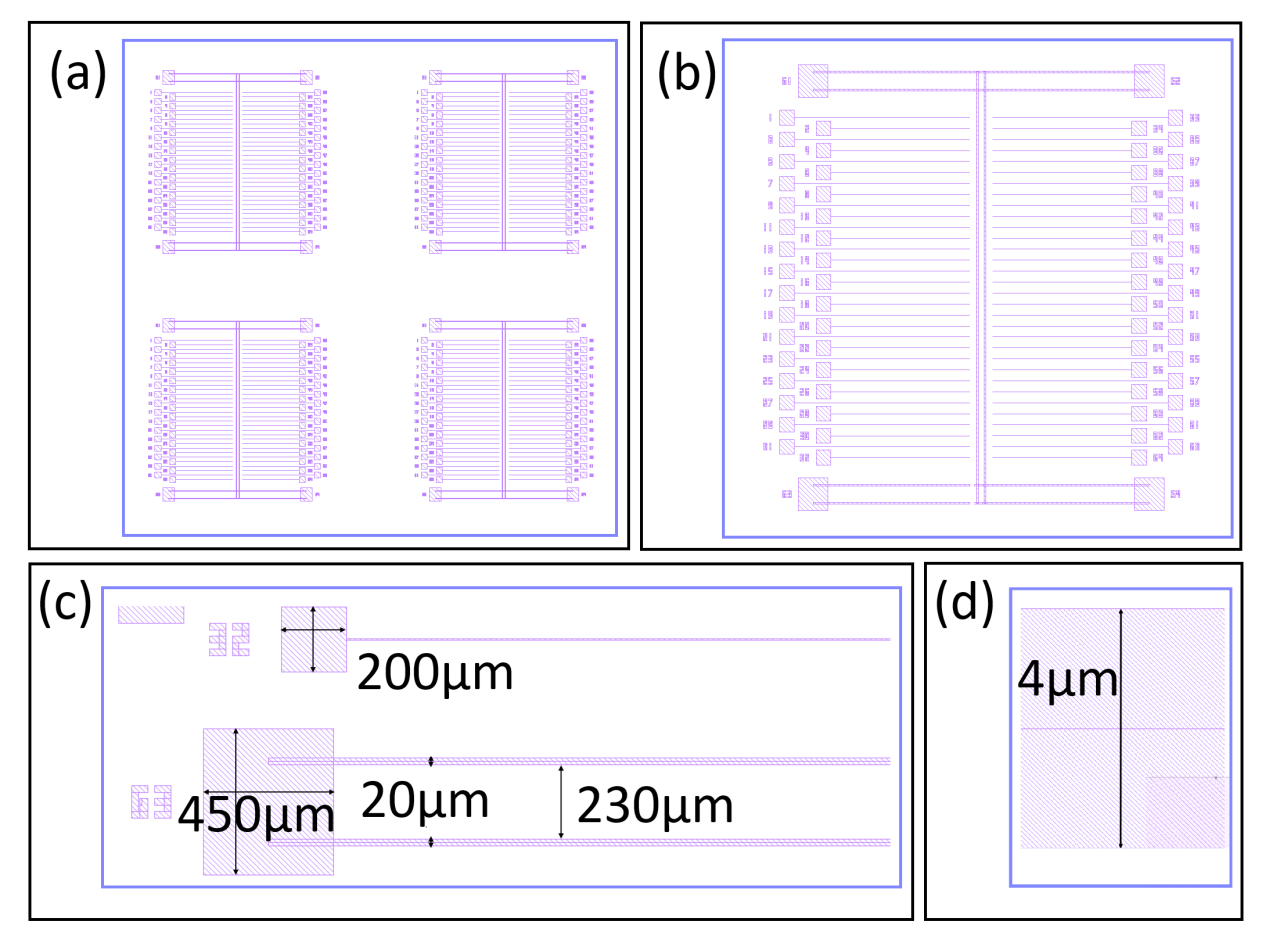
Metal deposition pattern. (**a**) Full-chip metal deposition pattern showing four identical devices. (**b**) Zoomed-in view of one device showing 64 metal traces and four additional bonding pads in the corners for electrical contact to the graphene. (**c**) Zoomed-in view of the graphene contact bonding pad and traces along with one trench. (**d**) Zoomed-in view of metal trace inside trench with its dimension.

The third step in our process is to create our metal deposition pattern as shown in Figure 9a. This pattern creates four devices in parallel. A zoomed-in view of one device is shown in Figure 9b. Here it is easy to see the 64 trenches with bonding pads at the end. We label each pad for easy tracking and communication purposes. Notice we also added four larger bonding pads at the outer corners of the device and connected these to each other with double traces. These will be used for electrical contact to the graphene. A zoomed-in view of our graphene electrical contact trace and one trench is shown in Figure 9c. The various dimensions are shown here as well. A zoomed-in view of the metal pattern inside the trench is shown with its dimension in Figure 9d. With this design we have one graphene electrical contact, but we have four bonding pads to choose from for both redundancy and convenience. In addition, each trench, which becomes the fixed side of a variable capacitor, has one bonding pad giving us 64 individually addressable variable capacitors with a common graphene connection. Of course, we can easily connect all 64 capacitors together in parallel to produce a single large capacitance capacitor. For that matter, once wire bonded, each package can be connected in parallel with other packages, as well. Finally, the thin trench lines used here also allow us to create a new pattern with 10 times more trenches, if desired.

#### 3.3.2. Metal Deposition Process

**Figure 10:**
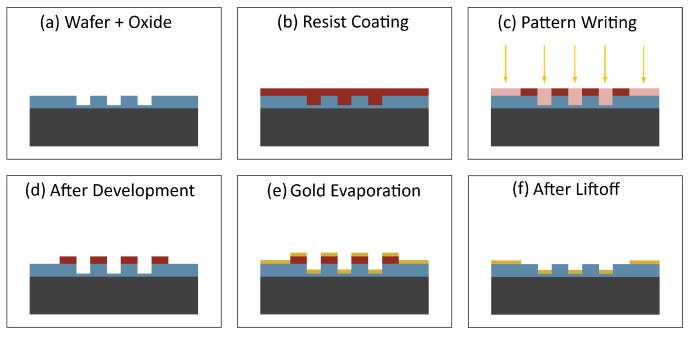
Metal deposition process. (**a**) Clean chip, (**b**) add photoresist, (**c**) write pattern, (**d**) develop, (**e**) deposit chromium and gold, and (**f**) lift-off unexposed photoresist.

An illustration of our metal deposition process is shown in Figure 10. The process is nearly identical to our alignment marker process presented earlier. This time metal will be deposited at the bottom of the etched trenches as well as on the top oxide surface. The process begins with a clean and dry chip as shown in Figure 10a. A photoresist is added as shown in Figure 10b. Our metal deposition pattern is written as shown in Figure 10c. After development, the resulting chip is as shown in Figure 10d. Next 5 nm of chromium followed by 95 nm of gold in deposited as shown in Figure 10e. We require more than 50 nm of gold for the wire bonder to work, but we can adjust this overall thickness to set the actual stand-off distance between the flexible graphene and the fixed metal electrode below it in the trench. Finally, lift-off is performed leaving only our metal pattern on top and in the trenches, as shown in Figure 10f. Once the metal deposition is completed we dice the chip into four identical devices. The final device measures approximately 8 mm by 8 mm and fits inside our device package.

#### 3.3.3. Metal Deposition Characterization

**Figure 11:**
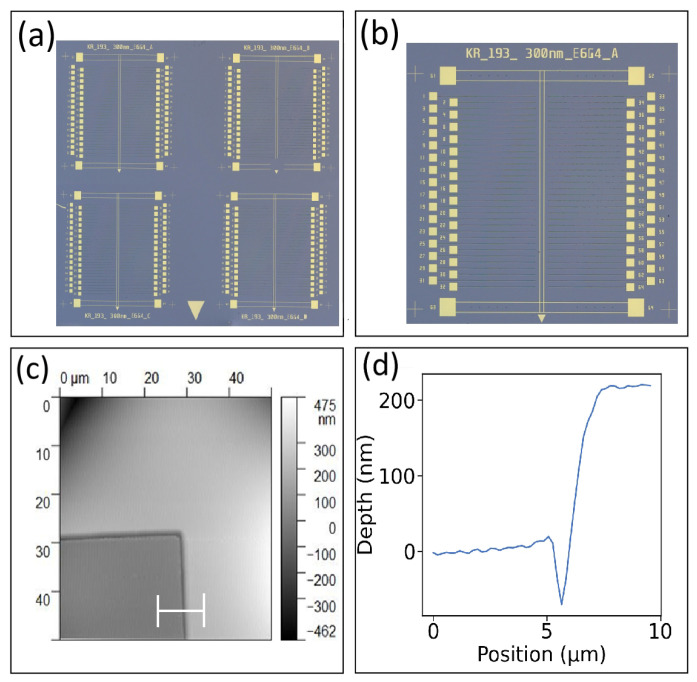
Metal deposition characterization. (**a**) Optical image of one chip showing four devices. (**b**) Zoomed-in optical image of one device and showing the top-layer double metal trace where graphene will rest and make electrical contact. (**c**) AFM image of one bonding pad inside an etched trench. (**d**) AFM line profile showing the distance between the top of the gold bonding pad and the top surface where graphene rests.

An optical image of our chip after metal deposition is shown in Figure 11a. A zoomed-in optical image of a single device measuring about 8 mm × 8 mm with 64 trenches and the two metal traces that graphene will rest on is shown in Figure 11b. An AFM image of a metal bonding pad inside the etched trench is shown in Figure 11c. A line profile taken from the AFM image is shown in Figure 11d. From the line profile, the surface of the metal bonding pad is about 200 μm below the top of the device where the graphene will rest. Of course, this same metal thickness is also on the top metal traces and used for wire bonding and graphene contacts.

### 3.4. Graphene Transfer

#### 3.4.1. Graphene Transfer Size and Placement Location

**Figure 12:**
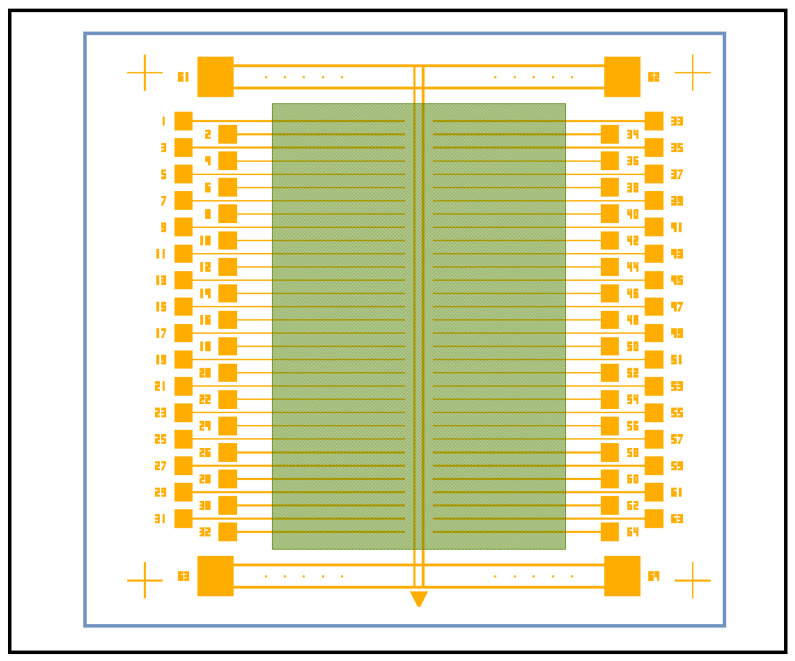
Placement plan for our 3 mm × 5 mm graphene on a single 8 mm × 8 mm device.

The fourth step in our process is to create the size and placement location for the graphene, as shown in Figure 12. The image shows our final device pattern with all the details. Notice the 64 trenches, the four outer crosshairs, the four outer bonding pads with the interconnecting double traces, the array of small square deep wells used for AFM depth profiling, and finally the green semitransparent graphene sheet that measures 3 mm × 5 mm. Notice the graphene partially covers all 64 trenches, simultaneously rests on the double metal trace down the center, yet avoids contacting any of the bonding pads. Our wire bonder only bonds to gold. If graphene covers the bonding pad, the wire bonder will fail to attach its wire to the bonding pad.

#### 3.4.2. Graphene Transfer Process

**Figure 13:**
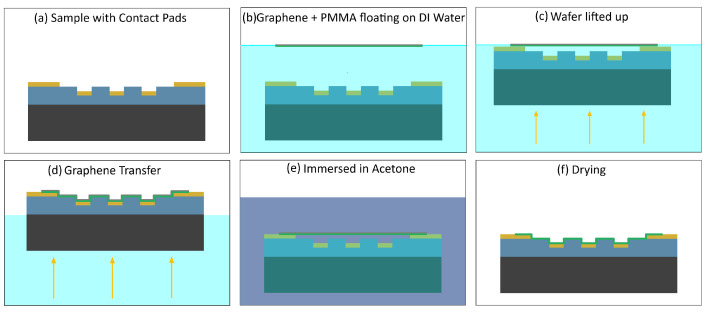
Graphene transfer process. (**a**) Clean device, (**b**) float PMMA–graphene in DI water and align device underneath, (**c**) lift device with PMMA–graphene out of the DI water, (**d**) dry the device overnight, (**e**) for PMMA removal immerse in acetone for 60 min, and (**f**) lift device with pure graphene out of the acetone and dry.

An illustration of our graphene transfer process is shown in Figure 13. The process begins with a clean chip as illustrated in Figure 13a. We use the same cleaning procedure as discussed before. We cut our commercially available PMMA–graphene to size and place it in DI water where it floats, as shown in Figure 13b. Our device is placed under the PMMA–graphene and precisely aligned using tweezers as shown in Figure 13c. At this stage, the device with the PMMA–graphene is lifted out of the water and allowed to dry overnight without heating, as shown in Figure 13d. We can choose to leave the PMMA attached to the graphene or remove it before further processing. Both types of devices are useful. Pure graphene is more flexible and PMMA–graphene is thicker and more massive. To remove the PMMA, we place the entire device into acetone for 60 min as illustrated in Figure 13e. Once the device is removed from the acetone the PMMA is no longer there, as shown in Figure 13f. In the end, we make two types of devices. The first type makes suspended PMMA–graphene capacitors and the other type makes suspended pure graphene capacitors.

#### 3.4.3. Graphene Transfer Characterization

**Figure 14:**
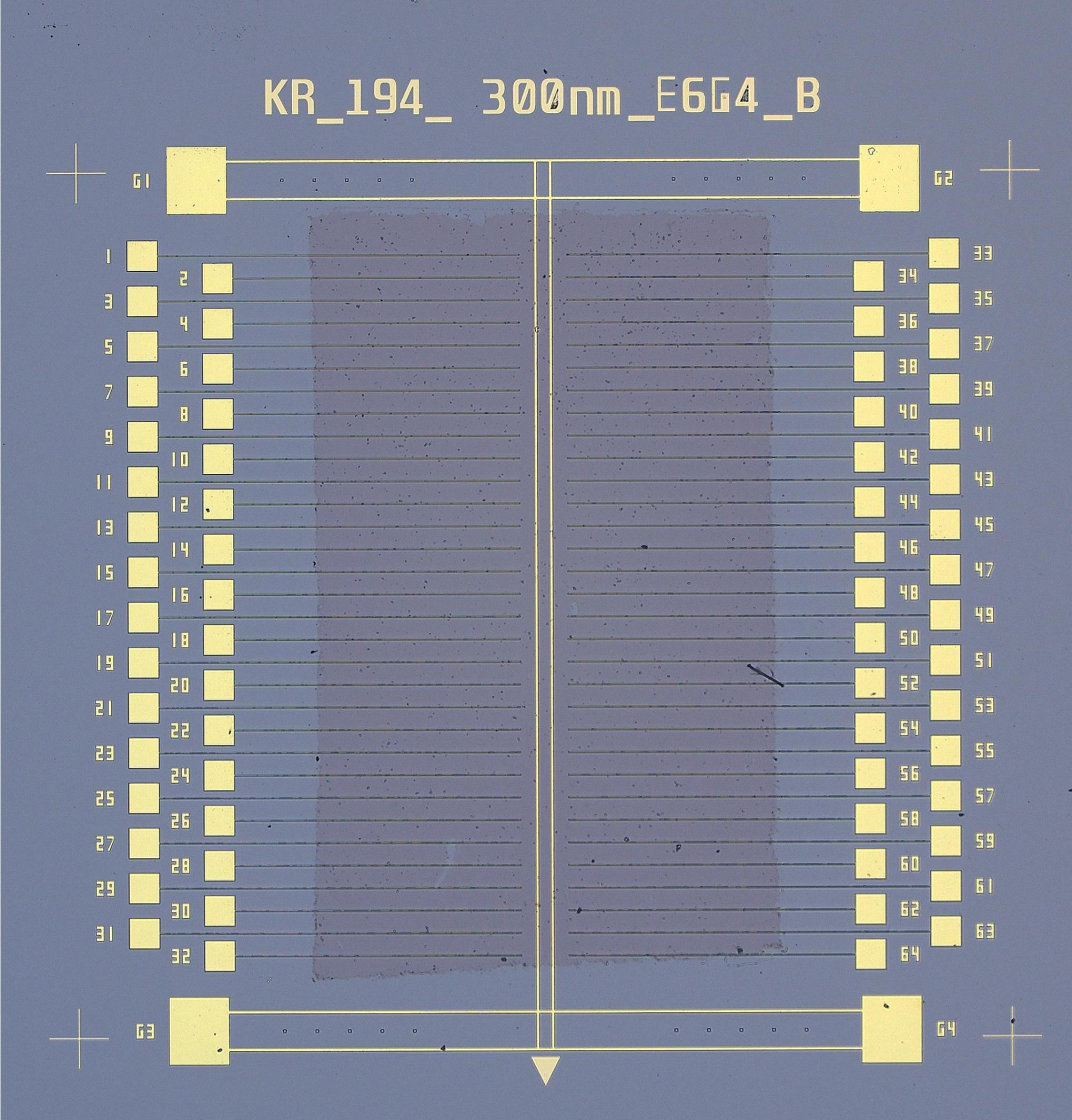
Optical image showing the size and placement of graphene sheet on one device.

An optical image of a single device measuring about 8 mm × 8 mm with pure graphene measure 3 mm × 5 mm on top is shown in Figure 14. The graphene partially covers most of the trenches. The graphene naturally rests on the double metal trace down the center, but does not touch any of the bonding pads.

### 3.5. Graphene Etch and Suspension

#### 3.5.1. Graphene Etch Pattern

**Figure 15:**
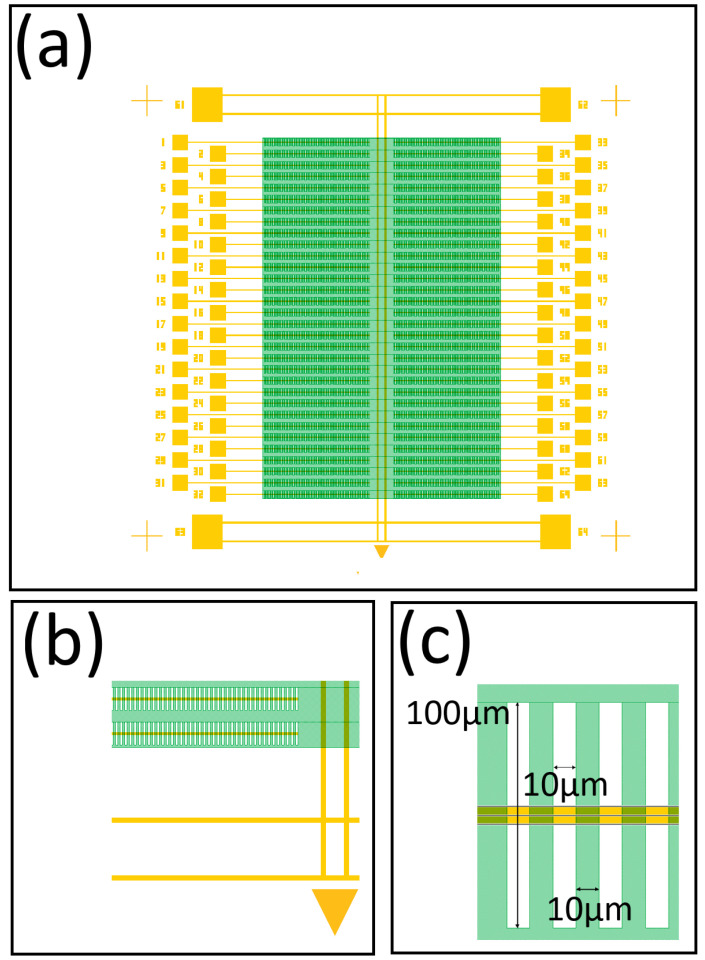
Graphene etch design pattern. (**a**) Device design with a 3 mm × 5 mm graphene sheet partially covering the trenches. (**b**) A zoomed-in view of the two end trenches with graphene shown. (**c**) A zoomed-in view showing the repeated pattern with areas of alternating graphene and removed graphene along the trench.

The fifth and final step in our process is to create a graphene etch pattern to aid suspension as shown in Figure 15a. A zoomed-in view of the graphene bonding pad and the adjacent trench with graphene is shown in Figure 15b. A zoomed-in view of a small section of graphene over a trench is shown in Figure 15c. At this scale it is easiest to see the graphene etch pattern. Notice we remove half the graphene covering the trenches using a 10 μm wide by 100 μm tall rectangle. The pattern is repeated every 20 μm. The array of 10 μm wide holes directly above the trenches assists with removing fluid from the trenches to aid the suspension of the adjacent 10 μm wide graphene [69].

#### 3.5.2. Graphene Etch Process

**Figure 16:**
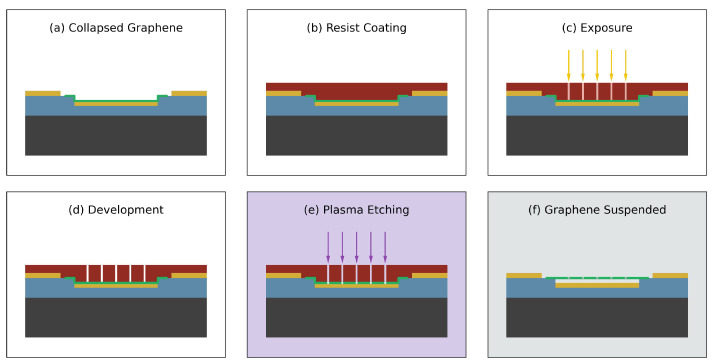
Graphene etch process. (**a**) Clean device with graphene, (**b**) add photoresist, (**c**) write pattern, (**d**) develop, (**e**) oxygen plasma etch, and (**f**) lift-off unexposed photoresist and place final device in IPA.

An illustration of our graphene etch process is shown in Figure 16. The process begins with a dry device with the graphene touching both the oxide surface and the gold surface, as shown in Figure 16a. Photoresist is added just like earlier, as shown in Figure 16b. Our graphene etch pattern is written as shown in Figure 16c. After development the resulting device is shown in Figure 16d. Next the device is etched using an oxygen plasma, as shown in Figure 16e. This step is carried out in 1 min time increments until the graphene is removed as observed under an optical microscope. Finally, lift-off is performed using the same procedure as earlier, followed by soaking the device in IPA, as shown in Figure 16f.

#### 3.5.3. Graphene Etch Characterization

**Figure 17:**
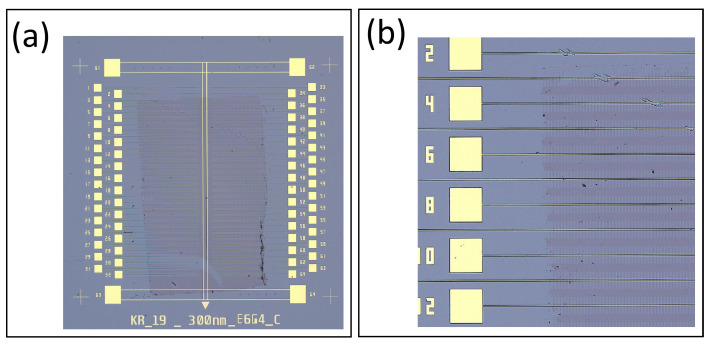
Graphene etch characterization. (**a**) Optical image of one device with graphene, and (**b**) zoomed-in view of 11 trenches showing graphene. Notice along each trench the graphene is patterned, and this shows that half of the graphene was removed.

An optical image of the device after oxygen plasma etching is shown in Figure 17a. A zoomed-in view of 11 trenches is shown in Figure 17b. At this scale it is easier to see the graphene has been patterned along the trenches. Essentially half the graphene was removed using a 20 μm wide repeated pattern. With half the graphene removed, the device in IPA solution is placed in our critical point dryer. This will dry the sample and leave the graphene suspended, after which the devices are wire bonded into a standard breadboard compatible package. Making the graphene freestanding was a very complex problem to solve. Initial suspension occurs because graphene floats on the surface of liquid IPA. As the liquid evaporates the graphene stays on its surface even as the level drops. Once the liquid surface reaches the bottom of the trench the graphene sticks to the gold surface at the bottom. To avoid this, we use a critical point dryer. However, the liquid IPA must be replaced with liquid $CO_{2}$ in a very short time period for the critical point dryer to work. The arrays of holes cut into the graphene every 10 μm allows for rapid fluid exchange.

### 3.6. Graphene Variable Capacitance

**Figure 18:**
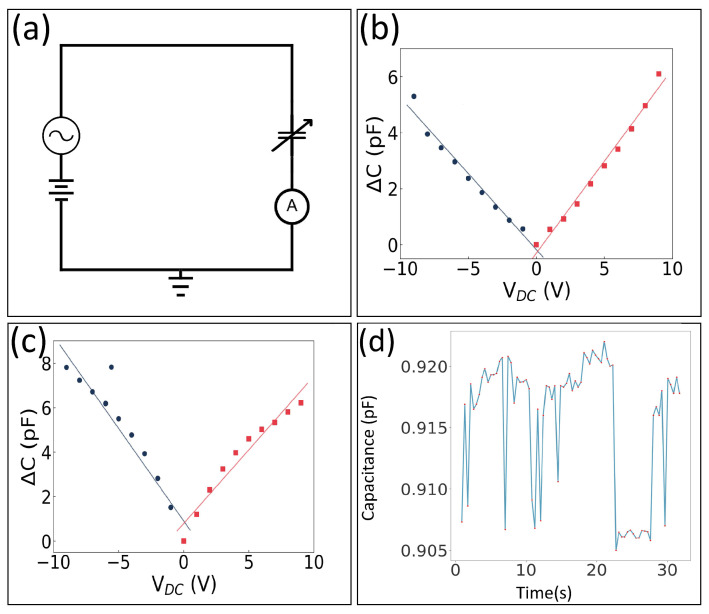
Graphene variable capacitance. (**a**) Electrical schematic of measurement setup. (**b**) Change in capacitance of a single trench for our pure graphene device as a function of applied DC bias voltage. (**c**) Change in capacitance of a single trench for our PMMA–graphene device as a function of applied DC bias voltage. (**d**) Time-dependent capacitance measurements showing random fluctuations.

In order to confirm the graphene over the trench forms a variable capacitor, we test the structures using the circuit schematic shown in Figure 18a. The setup uses an AC voltage source with a DC voltage offset connected to a single graphene variable capacitor and an AC ammeter. We set the AC voltage frequency to 100 kHz with an amplitude of 500 mV. A plot of the change in capacitance with applied DC bias voltage for pure graphene device is shown in Figure 18b. Notice the capacitance increases by about 5 pF when either a positive or negative bias voltage is applied. This response indicates that the graphene is being pulled toward the fixed electrode due to the electrostatic force, which is attractive for both positive and negative biases. A plot of the change in capacitance with an applied DC bias voltage for a single PMMA–graphene device is shown in Figure 18c. Notice the capacitance of the PMMA–graphene responds similarly and increases by about 8 pF when either a positive or negative bias voltage is applied. Using a sensitive capacitance meter we also measure the capacitance of a single trench in time as shown in Figure 18d. Here the capacitance is about 1 pF with clear fluctuations in time.

The AC capacitance of the variable capacitor was calculated from

$$C\;=\;\frac{I}{f\times \pi \times V_{\mathrm{pp}}},$$

where *I* is the measured AC current, *f* is the drive frequency, and $V_{\mathrm{pp}}$ is the peak-to-peak AC voltage. The DC offset voltage was swept from 0 to +9 V and from 0 to −9 V to characterize both the PMMA–graphene devices and the pure graphene devices.

We use an ultra-precise capacitance meter to make these measurements. It uses probes and a three-wire method to shield the leads, and as a result it is capable of measuring the capacitance down to the attoFarad level. For the wire bonded samples, there is parasitic capacitance; however, from the formula presented earlier, the current is only due to the changing capacitance in time. The results presented in Figure 18 are for a single trench. One can wire all 64 graphene variable capacitors together in a single package to boost the signal.

## 4. Conclusions

In this study, we presented a detailed fabrication process for producing an array of graphene-based variable capacitors for varying capacitance applications. Starting with a 100 mm diameter silicon wafer with a thermal oxide layer, thousands of variable capacitors were made, with 64 individually accessible graphene variable capacitors per packaged device. First, we diced the 100 mm wafer to a size that allowed us to produce four devices in parallel, then we added metallic alignment markers, followed by etching trenches into the oxide surface, depositing metal inside the trenches, and placing multilayer graphene over the trenches. Using oxygen plasma etching we patterned the graphene and suspended it using a critical point dryer. We successfully suspended both pure graphene and PMMA–graphene. The variable capacitors were wire bonded into a 28-pin package and tested. A single graphene suspension has capacitance variations of about 5 pF as a DC bias voltage is applied and forces the graphene closer to the fixed electrode below it.

## Data Availability

The original contributions presented in this study are included in the article. Further inquiries can be directed to the corresponding author.

## References

[1] Cook B.W., Berny A., Molnar A., Lanzisera S., Pister K.S. (2006). An Ultra-Low Power 2.4GHz RF Transceiver for Wireless Sensor Networks in 0.13/spl mu/m CMOS with 400mV Supply and an Integrated Passive RX Front-End. IEEE J. Solid-State Circuits.

[2] Law M.K., Bermak A., Luong H.C. (2010). A Sub-μW embedded CMOS temperature sensor for RFID food monitoring application. IEEE J. Solid-State Circuits.

[3] Zhang X., Jiang H., Zhang L., Zhang C., Wang Z., Chen X. (2009). An energy-efficient ASIC for wireless body sensor networks in medical applications. IEEE Trans. Biomed. Circuits Syst..

[4] Zhang Y., Zhang F., Shakhsheer Y., Silver J.D., Klinefelter A., Nagaraju M., Boley J., Pandey J., Shrivastava A., Carlson E.J. (2012). A batteryless 19 μW MICS/ISM-band energy harvesting body sensor node SoC for ExG applications. IEEE J. Solid-State Circuits.

[5] Hanson S., Seok M., Lin Y.S., Foo Z., Kim D., Lee Y., Liu N., Sylvester D., Blaauw D. (2009). A low-voltage processor for sensing applications with picowatt standby mode. IEEE J. Solid-State Circuits.

[6] Moser A., Erd M., Kostic M., Cobry K., Kroener M., Woias P. (2012). Thermoelectric energy harvesting from transient ambient temperature gradients. J. Electron. Mater..

[7] Liu Y., Riba J.R., Moreno-Eguilaz M., Sanllehi J. (2023). Application of thermoelectric generators for low-temperature-gradient energy harvesting. Appl. Sci..

[8] Roundy S., Wright P.K., Rabaey J. (2003). A study of low level vibrations as a power source for wireless sensor nodes. Comput. Commun..

[9] Amirtharajah R., Chandrakasan A.P. (2002). Self-powered signal processing using vibration-based power generation. IEEE J. Solid-State Circuits.

[10] Sun Y., Welch G.C., Leong W.L., Takacs C.J., Bazan G.C., Heeger A.J. (2012). Solution-processed small-molecule solar cells with 6.7% efficiency. Nat. Mater..

[11] Hösel M., Dam H.F., Krebs F.C. (2015). Development of lab-to-fab production equipment across several length scales for printed energy technologies, including solar cells. Energy Technol..

[12] Philp S.F. (1977). The Vacuum-Insulated, Varying-Capacitance Machine. IEEE Trans. Electr. Insul..

[13] Mitcheson P.D., Green T.C., Yeatman E.M., Holmes A.S. (2004). Architectures for vibration-driven micropower generators. J. Microelectromech. Syst..

[14] Yang B., Lee C., Kee W.L., Lim S.P. (2010). Hybrid energy harvester based on piezoelectric and electromagnetic mechanisms. J. Micro/Nanolithogr. MEMS MOEMS.

[15] Iqbal M., Nauman M.M., Khan F.U., Abas P.E., Cheok Q., Iqbal A., Aissa B. (2021). Vibration-based piezoelectric, electromagnetic, and hybrid energy harvesters for microsystems applications: A contributed review. Int. J. Energy Res..

[16] Novoselov K.S., Geim A.K., Morozov S.V., Jiang D.E., Zhang Y., Dubonos S.V., Grigorieva I.V., Frisov A.A. (2004). Electric field effects in atomically thin carbon films. Science.

[17] Nair R.R., Blake P., Grigorenko A.N., Novoselov K.S., Booth T.J., Stauber T., Peres N.M., Geim A.K. (2008). Fine structure constant defines visual transparency of graphene. Science.

[18] Wassei J.K., Kaner R.B. (2010). Graphene, a promising transparent conductor. Mater. Today.

[19] Xu Y., Liu J. (2016). Graphene as transparent electrodes: Fabrication and new emerging applications. Small.

[20] Chae S., Jang S., Choi W.J., Kim Y.S., Chang H., Lee T.I., Lee J.O. (2017). Lattice transparency of graphene. Nano Lett..

[21] Shao T., Wen B., Melnik R., Yao S., Kawazoe Y., Tian Y. (2012). Temperature dependent elastic constants and ultimate tensile strength of graphene and graphyne. J. Chem. Phys..

[22] Jiang J.W., Wang J.S., Li B. (2009). Young’s modulus of graphene: A molecular dynamics study. Phys. Rev. Condens. Matter Mater. Phys..

[23] Lee J.U., Yoon D., Cheong H. (2012). Estimation of Young’s modulus of graphene by Raman spectroscopy. Nano Lett..

[24] Tan X., Wu J., Zhang K., Peng X., Sun L., Zhong J. (2013). Nanoindentation models and Young’s modulus of monolayer graphene: A molecular dynamics study. Appl. Phys. Lett..

[25] Memarian F., Fereidoon A., Ganji M.D. (2015). Graphene Young’s modulus: Molecular mechanics and DFT treatments. Superlattices Microstruct..

[26] Morozov S.V., Novoselov K.S., Katsnelson M.I., Schedin F., Elias D.C., Jaszczak J.A., Geim A.K. (2008). Giant intrinsic carrier mobilities in graphene and its bilayer. Phys. Rev. Lett..

[27] Kashuba A.B. (2008). Conductivity of defectless graphene. Phys. Rev. B.

[28] Klimchitskaya G.L., Mostepanenko V.M. (2016). Conductivity of pure graphene: Theoretical approach using the polarization tensor. Phys. Rev. B.

[29] Wang Y., Huang Y., Song Y., Zhang X., Ma Y., Liang J., Chen Y. (2009). Room-temperature ferromagnetism of graphene. Nano Lett..

[30] Peres N.M.R., Guinea F., Neto A.H.C. (2005). Coulomb interactions and ferromagnetism in pure and doped graphene. Phys. Rev. B.

[31] Dong J., Zhang L., Ding F. (2019). Kinetics of graphene and 2D materials growth. Adv. Mater..

[32] Huang M., Ruoff R.S. (2020). Growth of single-layer and multilayer graphene on Cu/Ni alloy substrates. Acc. Chem. Res..

[33] Lee J., Novoselov K.S., Shin H.S. (2011). Interaction between metal and graphene: Dependence on the layer number of graphene. ACS Nano.

[34] Wang H., Wang Y., Cao X., Feng M., Lan G. (2009). Vibrational properties of graphene and graphene layers. J. Raman Spectrosc..

[35] Sze S.M., Li Y., Ng K.K. (2021). Physics of Semiconductor Devices.

[36] Song Y., Li X., Mackin C., Zhang X., Fang W., Palacios T., Zhu H., Kong J. (2015). Role of interfacial oxide in high-efficiency graphene-silicon Schottky barrier solar cells. Nano Lett..

[37] Rehman M.A., Roy S.B., Akhtar I., Bhopal M.F., Choi W., Nazir G., Khan M.F., Kumar S., Eom J., Chun S.H. (2019). Thickness-dependent efficiency of directly grown graphene based solar cells. Carbon.

[38] Choi Y., Lee J., Seo J., Jung S., Kim U., Park H. (2017). The effect of the graphene integration process on the performance of graphene-based Schottky junction solar cells. J. Mater. Chem..

[39] Gnisci A., Faggio G., Lancellotti L., Messina G., Carotenuto R., Bobeico E., Veneri P.D., Capasso A., Dikonimos T., Lisi N. (2019). The role of graphene-based derivative as interfacial layer in graphene/n-Si Schottky barrier solar cells. Phys. Status Solidi A.

[40] Jehad A.K., Yurddaskal M., Gunes F., Zafer C., Kocabas K. (2021). Investigation of graphene-based Schottky junction solar cell with heavy-doped silicon. J. Mater. Sci. Mater. Electron..

[41] Fallahazad P., Naderi N., Eshraghi M.J. (2020). Improved photovoltaic performance of graphene-based solar cells on textured silicon substrate. J. Alloys Compd..

[42] Arefinia Z., Asgari A. (2014). A new modeling approach for graphene-based silicon nanowire Schottky junction solar cells. J. Renew. Sustain. Energy.

[43] Ju S., Liang B., Wang J.Z., Shi Y., Li S.L. (2018). Graphene/silicon Schottky solar cells: Technical strategies for performance optimization. Opt. Commun..

[44] Rahman S.M., Kabir M.R., Amin T.B., Mangum J.M., Ashaduzzaman, Thibado P.M. (2024). Array of Graphene Solar Cells on 100 mm Silicon Wafers for Power Systems. Energies.

[45] Ashaduzzaman, Rahman S.M., Kabir M.R., Mangum J.M., Do H., Carichner G., Blaauw D., Thibado P.M. (2025). Array of mini-graphene-silicon solar cells intermittently recharges storage capacitors powering a temperature sensor. J. Vac. Sci. Technol..

[46] Mermin N.D., Wagner H. (1966). Absence of ferromagnetism in one-or-two dimensional isotropic Heisenberg models. Phys. Rev. Lett..

[47] Bonilla L.L., Carpio A. (2012). Ripples in a graphene membrane coupled to Glauber spins. J. Stat. Mech. Theory Exp..

[48] Ruiz-Garcia M., Bonilla L.L., Prados A. (2015). Ripples in hexagonal lattices of atoms coupled to Glauber spins. J. Stat. Mech. Theory Exp..

[49] Fasolino A., Los J.H., Katsnelson M.I. (2007). Intrinsic ripples in graphene. Nat. Mater..

[50] Hašik J., Tosatti E., Martoňák R. (2018). Quantum and classical ripples in graphene. Phys. Rev..

[51] Talla J.A., Ahmad M.S. (2022). Structural and electronic properties of rippled graphene monolayer: Density functional theory. J. Electron. Mater..

[52] Cavallucci T., Tozzini V. (2016). Multistable rippling of graphene on SiC: A density functional theory study. J. Phys. Chem..

[53] Yamaletdir nov R.D., Ivakhnenko O.V., Sedelnikova O.V., Shevchenko S.N., Pershin Y.V. (2018). Snap-through transition of buckled graphene membranes for memcapacitor applications. Sci. Rep..

[54] Xiang Y., Shen H.S. (2016). Compressive buckling of rippled graphene via molecular dynamics simulations. Int. J. Struct. Stab. Dyn..

[55] Breitwieser R., Hu Y.C., Chao Y.C., Tzeng Y.R., Liou S.C., Lin K.C., Chen C.W., Pai W.W. (2017). Investigating ultraflexible freestanding graphene by scanning tunneling microscopy and spectroscopy. Phys. Rev. B.

[56] Zan R., Muryn C., Bangert U., Mattocks P., Wincott P., Vaughan D., Li X., Colombo L., Ruoff R.S., Hamilton B. (2012). Scanning tunnelling microscopy of suspended graphene. Nanoscale.

[57] Lindahl N., Midtvedt D., Svensson J., Nerushev O.A., Lindvall N., Isacsson A., Campbell E.E. (2012). Determination of the bending rigidity of graphene via electrostatic actuation of buckled membranes. Nano Lett..

[58] Scharfenberg S., Mansukhani N., Chiavlo C., Weaver R.L., Mason N. (2012). Observation of a snap-through instability in graphene. Appl. Phys. Lett..

[59] Meyer J.C., Geim A.K., Katznelson M.I., Novoselov K.S., Booth T.J., Roth S. (2007). The structure of suspended graphene sheets. Nature.

[60] Xu P., Neek-Amal M., Barber S.D., Schoelz J.K., Ackerman M.L., Thibado P.M., Sadeghi A., Peeters F.M. (2014). Unusual ultralow frequency fluctuations in freestanding graphene. Nat. Commun..

[61] Ackerman M.L., Kumar P., Neek-Amal M., Thibado P.M., Peeters F.M., Singh S.P. (2016). Anomalous Dynamical Behavior of Freestanding Graphene Membranes. Phys. Rev. Lett..

[62] Mangum J.M., Kabir M.R., Amin T.B., Rahman S.M., Ashaduzzaman, Thibado P.M. (2025). Spectrum Analysis of Thermally Driven Curvature Inversion in Strained Graphene Ripples for Energy Conversion Applications via Molecular Dynamics. Nanomaterials.

[63] Shearer C.J., Slattery A.D., Stapleton A.J., Shapter J.G., Gibson C.T. (2016). Accurate thickness measurement of graphene. Nanotechnology.

[64] Kumar V., Kumar A., Lee D.J., Park S.S. (2021). Estimation of number of graphene layers using different methods: A focused review. Materials.

[65] Ni Z.H., Wang H.M., Kasim J., Fan H.M., Yu T., Wu Y.H., Feng Y.P., Shen Z.X. (2007). Graphene thickness determination using reflection and contrast spectroscopy. Nano Lett..

[66] Rickhaus P., Liu M.H., Kurpas M., Kurzmann A., Lee Y., Overweg H., Eich M., Pisoni R., Taniguchi T., Watanabe K. (2020). The electronic thickness of graphene. Sci. Adv..

[67] Maragliano C., Glia A., Stefancich M., Chiesa M. (2014). Effective AFM cantilever tip size: Methods for in-situ determination. Meas. Sci. Technol..

[68] AbdelGhany M., Ledwosinska E., Szkopek T. (2012). Theory of the suspended graphene varactor. Appl. Phys. Lett..

[69] AbdelGhany M., Mahvash F., Mukhopadhyay M., Favron A., Martel R., Siaj M., Szkopek T. (2016). Suspended graphene variable capacitor. 2D Mater..

[70] Yang J., Wei D., Tang L., Song X., Luo W., Chu J., Gao T., Shi H., Du C. (2025). Wearable temperature sensor based on graphene nanowalls. RSC Adv..

[71] Davaji B., Cho H.D., Malakoutian M., Lee J.K., Panin G., Kang T.W., Lee C.H. (2017). A patterned single layer graphene resistance temperature sensor. Sci. Rep..

[72] Wang Y., Wang Y., Zhang P., Liu F., Luo S. (2018). Laser induced freestanding graphene papers: A new route of scalable fabrication with tunable morphologies and properties for multifunctional devices and structures. Small.

[73] Wang Y., Niu Z., Chen J., Zhai Y., Xu Y., Luo S. (2019). Freestanding laser induced graphene paper based liquid sensors. Carbon.

[74] Yavari F., Koratkar N. (2012). Graphene-based chemical sensors. J. Phys. Chem. Lett..

[75] Zhang M., Halder A., Hou C., Ulstrup J., Chi Q. (2016). Free-standing and flexible graphene papers as disposable non-enzymatic electrochemical sensors. Bioelectrochemistry.

[76] Thibado P.M., Kumar P., Singh S., Ruiz-Garcia M., Lasanta A., Bonilla L.L. (2020). Fluctuation-induced current from freestanding graphene. Phys. Rev..

[77] Gikunda M.N., Harerimana F., Mangum J.M., Rahman S., Thompson J.P., Harris C.T., Churchill H.O., Thibado P.M. (2022). Array of graphene variable capacitors on 100 mm silicon wafers for vibration-based applications. Membranes.

[78] Durbin J., Mangum J.M., Gikunda M.N., Harerimana F., Amin T., Kumar P., Bonilla L.L., Thibado P.M. (2023). Freestanding graphene heat engine analyzed using stochastic thermodynamics. AIP Adv..

[79] Akbari S., Ghafarinia V., Larsen T., Parmar M., Villanueva L. (2020). Large Suspended Monolayer and Bilayer Graphene Membranes with Diameter up to 750 μm. Sci. Rep..

[80] Hu H., Liao B., Guo X., Hu D., Qiao X., Liu N., Liu R., Chen K., Bai B., Yang X. (2017). Large-Scale Suspended Graphene Used as a Transparent Substrate for Infrared Spectroscopy. Small.

[81] Chen Y., He S., Huang C., Huang C., Shih W., Chu C., Kong J., Li J., Su C. (2016). Ultra-large suspended graphene as a highly elastic membrane for capacitive pressure sensors. Nanoscale.

[82] Carvalho A., Fernandes A., Hassine M.B., Ferreira P., Fortunato E., Costa F. (2020). Millimeter-sized few-layer suspended graphene membranes. Appl. Mater. Today.

[83] Lukas S., Esteki A., Rademacher N., Jangra V., Gross M., Wang Z., Ngo H., Bäuscher M., Mackowiak P., Höppner K. (2024). High-Yield Large-Scale Suspended Graphene Membranes over Closed Cavities for Sensor Applications. ACS Nano.

[84] Ashaduzzaman, Mangum J.M., Rahman S.M., Amin T.B., Kabir M.R., Do H., Carichner G., Blaauw D., Thibado P.M. (2025). Low-Level Kinetic-Energy-Powered Temperature Sensing System. J. Low Power Electron. Appl..

